# Development of *Streptococcus pyogenes* pneumnonia and pleural empyema post-chickenpox infection in a 5-year-old child: A case report

**DOI:** 10.5339/qmj.2024.67

**Published:** 2024-11-11

**Authors:** Majed Abu Jaish, Mai Akila, Yazan AlHabil

**Affiliations:** 1 Department of Pediatrics, Arab Women’s Union Hospital, Nablus, Palestine; 2 Department of Human Medicine, Faculty of Medicine and Health Sciences, An-Najah National University, Nablus, Palestine *Email: yazanalhabil@gmail.com

**Keywords:** Pediatrics, chickenpox, pleural empyema, infection, varicella

## Abstract

**Background:**

The introduction of the varicella vaccine has led to a significant decrease in pediatric varicella-induced invasive *Streptococcus pyogenes* (group A streptococcal [GAS]) infections. However, the development of a pleural empyema following a chickenpox infection is a rare complication in pediatric patients.

**Case presentation:**

In this report, we present a 5-year-old male patient who presented to the emergency department with a deteriorating course two days after a chickenpox infection. The patient complained of high-grade documented fever, a congested throat, abdominal pain, shortness of breath, and most importantly, decreased air entry on the right side of the chest, along with the presence of crepitations. Such a deteriorated clinical picture suggested the presence of an infectious cause. The patient’s physical examination and radiological imaging provided evidence for the presence of lower right-sided lobar pneumonia. On the second day of hospitalization, the patient showed worsening respiratory distress, prompting further investigations that confirmed the development of a right-sided pleural empyema through radiological imaging. Pediatric surgery consultation was requested, and 500 cc of pus was drained following the insertion of a chest tube, which was later sent for analysis. The patient’s clinical picture improved significantly following this intervention. Due to the severity of his condition, the patient was transferred to the pediatric intensive care unit (PICU) for close monitoring. After one night in the PICU, during which his condition stabilized and oxygen therapy was gradually weaned off, the patient continued to improve on the general ward. Daily assessments and laboratory tests showed decreasing inflammatory markers and resolution of symptoms. Following three days of admission and confirmation of no underlying immunologic deficiency, the patient was discharged home with appropriate antibiotic therapy and follow-up instructions.

**Discussion:**

Similar cases have been sporadically documented in pediatric literature, with notable examples involving older patients. The pathophysiology involves complex immune interactions and virulence factors of GAS, contributing to severe outcomes such as pleural effusion.

**Conclusion:**

In this case, the 5-year-old patient experienced a severe progression from chickenpox to pleural empyema but ultimately improved following prompt medical intervention and chest tube drainage. The patient was discharged after a successful recovery, highlighting the efficacy of early recognition and treatment in managing such complications.

## 1. Introduction

Community-acquired pneumonia (CAP) in pediatric settings is caused by many pathogens, with *Streptococcus pneumonia* (SP) and *Streptococcus pyogenes* (group A streptococcal [GAS]) being the most common.^1^ GAS is responsible for a wide array of invasive infections. Current literature points to an increase of invasive GAS infections worldwide in the last decade, along with associated mortality, subsequent morbidity, and prolonged hospital stays.^1^ On the other hand, despite the introduction of anti-pneumococcal conjugated vaccines and subsequent decreased mortality rates in the last decade, SP remains the principal cause of CAP’s complications.^2,3^ Para-pneumonic effusion and pleural empyema are two of the uncommon complications.^2,3^

The association between varicella infections (chickenpox) and invasive GAS infections has been well-established in the literature. Laupland et al. reported a relative risk (RR) of 58 (95% CI: 40–85), indicating that children with chickenpox infection faced a significantly increased risk of acquiring invasive GAS infections, with approximately one in seven (15%) affected.^4^

The introduction of the varicella vaccine has been associated with a significant decline in pediatric varicella-induced invasive GAS hospitalizations.^5,6^ However, undergoing recent surgery, experiencing recent trauma, being a male, and having an underlying immunodeficiency disorder appear to increase the risk of invasive GAS infections.^7^ Invasive GAS infections encompass a range of conditions such as pneumonia, subcutaneous abscesses, osteoarticular infections, and mastoiditis. Among these, pneumonia in younger patients exhibiting elevated levels of C-reactive protein is associated with a heightened risk of admission to the pediatric intensive care unit (PICU).^8^

To the best of our knowledge, this is the first case report to address a significant knowledge gap by reporting the onset of GAS-induced pleural effusion following varicella infection in a 5-year-old Middle Eastern male patient with no underlying pathologies. Our institution does not require formal approval to publish case reports. We obtained both oral and written consent from the patient’s father.

## 2. Case Presentation

A 5-year-old male patient, weighing 20 kg (44 pounds) and up-to-date with the Palestinian national vaccination program, was in his usual state of health until about two days prior to his presentation. Initially, the patient exhibited a polymorphic skin rash on the trunk, diagnosed as a chickenpox infection at a private outpatient clinic. In response, the patient was discharged, and the management approach solely involved supportive care for this condition. However, one day later, the patient’s clinical picture deteriorated, where the patient developed a high-grade documented fever (39°C on axillary measurement), along with shortness of breath, productive cough, abdominal pain, and a couple of episodes of vomiting. This prompted the patient’s family to seek medical advice at the hospital’s emergency department.

**Figure 1.**
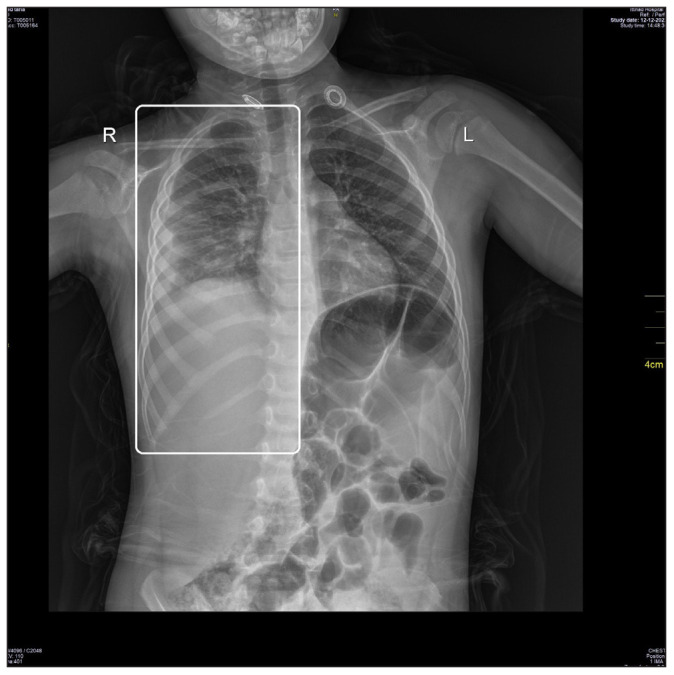
The patient’s initial X-ray image demonstrating a right-sided lower lobe pneumonia (white rectangle).

The patient’s physical examination showed an ill, irritable, feverish patient with generalized polymorphic skin lesions that were characteristic of a chickenpox infection. In addition, the patient’s examination disclosed a congested throat, shortness of breath, and generalized abdominal pain. Abdominal assessment demonstrated positive findings for abdominal rigidity, muscle guarding, and rebound tenderness. There was no lymph node enlargement. Additionally, the patient had significantly decreased air entry upon the examination of the right side of the chest, as well as the presence of crepitations. Following the patient’s physical examination, he was admitted to the hospital’s pediatric ward. The patient was administered oxygen through a face mask at a rate of 5 liters per minute, with an O_2_ saturation above 94%, which was gradually weaned off as the patient’s clinical picture improved. Furthermore, a chest X-ray was taken, which provided evidence of right lower-lobe pneumonia (Figure 1).

Therefore, the patient received a combination of ceftriaxone (70 mg/kg/day), vancomycin (15 mg/kg every 6 hours), and acyclovir (20 mg/kg/day). Ceftriaxone and vancomycin were initiated empirically in response to the severe clinical presentation and suspicion of a staphylococcal infection or potential resistance of certain organisms to cephalosporins. In addition, blood tests showed a white-blood-cell count of 10,000 cells/µL (normal range: 4,000–10,000 cells/µL), C-reactive protein of 300 mg/dL (normal range: < 1.38 mg/dL), and a procalcitonin level of 100 ng/mL (normal range: < 0.1 ng/mL). Finally, the patient’s kidney and liver function tests, along with the serum lytes were all within the normal ranges.

**Figure 2.**
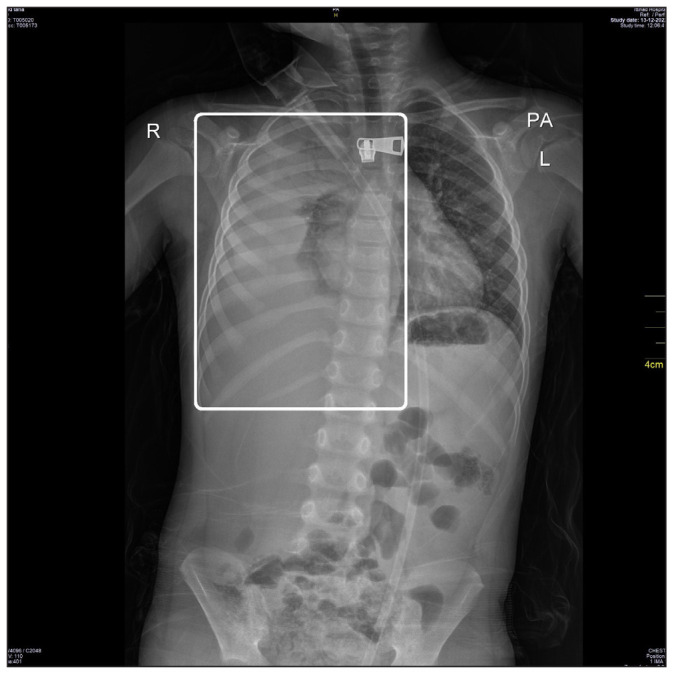
The patient’s second X-ray image demonstrating a right-sided pleural effusion (white rectangle).

On the second day of the patient’s hospitalization, he showed worsening respiratory distress, requiring oxygen therapy. Another round of oxygen via face mask at 5 liters per minute maintained his oxygen saturation above 94%. There was suspicion of effusion vs. the presence of loculated multiple abscesses. Therefore, another chest X-ray scan was taken for further confirmation, which provided evidence for the presence of a right-sided pleural effusion (Figure 2). Immediately afterward, a chest computed tomography (CT) scan without intravenous (IV) contrast and a chest ultrasound scan were performed, which provided evidence for the presence of a large amount of right pleural effusion, more than 300 cc. The potential association between pneumonia caused by SP and right-sided pleural empyema in the context of a chickenpox infection was investigated after conducting a molecular study on respiratory causes using a nasopharyngeal swab, which yielded negative results (Table 1). The diagnosis of chickenpox was established clinically, with the characteristic lesions being in the advanced stage of crusting upon examination. Due to the patient’s deteriorated clinical picture, later that day, the patient’s pharmaceutical management was changed to also include piperacillin-tazobactam (112.5 mg/kg every 6 hours). Additionally, the patient was started on oseltamivir (45 mg/kg every 12 hours).

The following day, a pediatric surgeon was consulted to participate in the management of this case. Under general anesthesia, the physician inserted a chest tube in the patient’s right chest, which yielded 500 cc of pus. The drained liquid was subsequently sent for analysis, culture, and cytology (Table 2). Following the procedure, the patient stayed in the PICU for one night on an O_2_ face mask at a rate of 5 liters per minute, with an O_2_ saturation above 94%.

The patient’s clinical picture improved after the drainage of the pleural effusion was performed. The patient’s fever subsided, and he started to tolerate oral intake with no reported abdominal pain. In addition, a gradual weaning off of oxygen was performed. A daily physical examination and follow-up C-reactive protein and procalcitonin count tests were performed for the remainder of the patient’s stay, which was for three days following his admission to the PICU, during which these parameters were decreasing gradually. Furthermore, due to the patient’s severe complications and initial deteriorated clinical picture, an immunoglobulin levels assay was performed to rule out any underlying immunological deficiencies. Fortunately, the patient’s assay levels were all within the normal ranges for his age. Following the three days of hospitalization and the subsequent improvement in the patient’s clinical picture, a four-week course of oral antibiotics was prescribed based on culture sensitivity results. This treatment included a daily dose of amoxicillin and clavulanic acid suspension (125 mg/5 mL) and clindamycin (10 mg/kg/day).

## 3. Discussion

Despite the rarity of pleural empyema caused by a GAS infection, there is limited documentation of such cases in the current literature. Pleural empyema following a varicella infection is mostly reported to present in adult patients, especially in the setting of acute lymphoblastic leukemia, chemotherapy, and other immunocompromised populations.^9,10^ Although developing varicella pneumonia is more common in adults, our case report suggests that it can also develop in pediatric populations.

Yamaguchi et al. described the development of pleural empyema post-varicella infection with the culture pointing towards an invasive GAS infection in a 21-month-old immunocompetent boy.^11^ These findings aligned seamlessly with the case we presented, considering the notably older age of our patient. Moreover, Mahto et al. described a comparable case to both ours and the aforementioned one. However, the patient featured in their case presentation was an 11-year-old male.^12^

To the best of our knowledge, these two aforementioned case reports stand as the only documented evidence of varicella-induced pleural effusion in pediatric populations. Lastly, it is notable that Carapetis et al. documented a case where an infant developed cardiac tamponade secondary to pericardial effusion, concomitant with the presence of a pleural effusion following a varicella infection.^13^

The pathophysiology of the association between chickenpox and SP pneumonia with pleural empyema involves intricate immune interactions. SP utilizes virulence factors, including the M-protein in its cell wall, to inhibit complement activation and evade phagocytosis. The administration of clindamycin effectively deactivates these virulence factors, making it a suitable choice in treatment plans.^14^ Additionally, the bacterium produces exotoxins capable of directly activating T cells, triggering a cytokine storm characterized by the release of inflammatory cytokines, including IL-1β.^15^ This heightened inflammatory response contributes to the severity of invasive GAS infections. Interestingly, Fujimura et al. report that in the setting of atopic dermatitis, switching of the CD4+ T cells may occur in individuals with varicella infections and altered humoral immunity.^14,15^ The detailed interplay of immune evasion, cytokine response, and potential adjustments highlights the complexity of the disease process in this infection.^16^

Laupland et al. report that a varicella infection significantly increases the risk of developing invasive GAS infections in children by 58 times, though the reason for this remains unclear. Additionally, children with both invasive GAS and recent chickenpox infections face a higher likelihood of developing necrotizing fasciitis (RR: 6.3; 95% CI: 1.8–22.3).^4^ In addition, Patel et al. provide robust evidence supporting the critical role of vaccination at 1 year of age, highlighting its efficacy in reducing the susceptibility to invasive GAS infections among pediatric populations (6). To the best of our knowledge, our case report marks the first documentation of GAS-induced pleural effusion following varicella infection in a 5-year-old Middle Eastern male patient without underlying pathologies. The limitations of our case report stem from the scarcity of available evidence in the current literature regarding the addressed subject.

## 4. Conclusions

Invasive GAS infections and their complications can manifest in pediatric patients following a chickenpox infection in a wide array of forms. Our case report presents a rare case of the development of *Streptococcus pyogenes* pneumonia and consequent pleural empyema in a 5-year-old patient following a chickenpox infection. Initially managed with supportive care for chickenpox, the patient experienced rapid deterioration with high fever, respiratory distress, and abdominal symptoms, leading to hospital admission. Diagnostic imaging revealed right lower-lobe pneumonia and a large pleural effusion. The patient received aggressive antibiotic therapy (ceftriaxone and vancomycin) and antiviral treatment (acyclovir). On the second day of hospitalization, a pediatric surgeon performed drainage of 500 cc of pleural pus. After one day in the PICU and three days on the ward, the patient improved significantly and was discharged with a full recovery expected. This case report is deemed worthy of sharing in hopes of providing a clinical blueprint for pediatric healthcare professionals to follow if they ever have to deal with such a rare occurrence. All in all, despite the rigid implementation of international vaccination programs, healthcare professionals must be aware of such a rare occurrence in pediatric patients.

## Acknowledgement

We acknowledge the patient and his family for allowing us to write this case report.

## Figures and Tables

**Table 1.** A molecular analysis (real-time polymerase chain reaction) of the patient’s nasopharyngeal swab (conducted on the second day of hospitalization).

**Parameter**	**Result**	**Interpretation**
Influenza Virus A	Negative	Viral RNA/DNA not detected under test conditions
Influenza Virus A (H1N1)	Negative	
Influenza Virus A (H3)	Negative	
SARS-CoV-2	Negative	

**Table 2.** Analysis of the drained fluid.

**Characteristics**	**Results**	**Reference range**
Color	Green	Not available
Appearance	Turbid	Not available
Glucose levels (mg/dl)	1.5	< 60
Protein levels (mg/dl)	4.7	< 3
White blood cells (cells/µl)	64,000	4,000–10,000
LDH (IU/L)	10,383	< 1,000
Neutrophil count (%)	Not available	Not available
Lymphocytes count (%)	Not available	Not available
Culture results	Moderate *Streptococcus pyogenes* (blood culture was negative)	Not available
Drug sensitivity test	Sensitive to: clindamycin and amoxicillin/clavulanic acid	Not available
